# Multiple-Locus Variable Number Tandem Repeat Analysis of *Staphylococcus Aureus*: Comparison with Pulsed-Field Gel Electrophoresis and *spa*-Typing

**DOI:** 10.1371/journal.pone.0005082

**Published:** 2009-04-03

**Authors:** Leo M. Schouls, Emile C. Spalburg, Martijn van Luit, Xander W. Huijsdens, Gerlinde N. Pluister, Marga G. van Santen-Verheuvel, Han G. J. van der Heide, Hajo Grundmann, Max E. O. C. Heck, Albert J. de Neeling

**Affiliations:** Laboratory for Infectious Diseases and Perinatal screening, National Institute for Public Health and the Environment, Bilthoven, the Netherlands; Columbia University, United States of America

## Abstract

**Background:**

Molecular typing of methicillin-resistant *Staphylococcus aureus* (MRSA) is required to study the routes and rates of transmission of this pathogen. Currently available typing techniques are either resource-intensive or have limited discriminatory ability. Multiple-locus variable number tandem repeat analysis (MLVA) may provide an alternative high throughput molecular typing tool with high epidemiological resolution.

**Methodology/Principal Findings:**

A new MLVA scheme for *S. aureus* was validated using 1681 *S. aureus* isolates collected from Dutch patients and 100 isolates from pigs. MLVA using 8 tandem repeat loci was performed in 2 multiplex PCRs and the fluorescently labeled PCR products were accurately sized on an automated DNA sequencer. The assessed number of repeats was used to create MLVA profiles consisting of strings of 8 integers that were used for categorical clustering. MLVA yielded 511 types that clustered into 11 distinct MLVA complexes which appeared to coincide with MLST clonal complexes. MLVA was at least as discriminatory as PFGE and twice as discriminatory as *spa*-sequence typing. There was considerable congruence between MLVA, *spa*-sequence typing and PFGE, at the MLVA complex level with group separation values of 95.1% and 89.2%. MLVA could not discriminate between pig-related MRSA strains isolated from humans and pigs, corroborating the high degree of relationship. MLVA was also superior in the grouping of MRSA isolates previously assigned to temporal-spatial clusters with indistinguishable SpaTypes, demonstrating its enhanced epidemiological usefulness.

**Conclusions:**

The MLVA described in this study is a high throughput, relatively low cost genotyping method for *S. aureus* that yields discrete and unambiguous data that can be used to assign biological meaningful genotypes and complexes and can be used for interlaboratory comparisons in network accessible databases. Results suggest that MLVA offsets the disadvantages of other high discriminatory typing approaches and represents a promising tool for hospital, national and international molecular epidemiology.

## Introduction

*Staphylococcus aureus* is an important bacterial pathogen that is associated with serious community-acquired and nosocomial diseases [1], [2]. Although it can cause a variety of clinical syndromes such as bacteremia, pneumonia, endocarditis, and deep abscess formation, *S. aureus* seems to be omnipresent and is more often than not carried without any clinical symptoms. Carriers may spread the pathogen infecting individuals who may develop disease. The introduction of methicillin has led to the rapid emergence of methicillin-resistant *S. aureus* (MRSA) and is posing a major clinical problem within hospitals worldwide [3]–[5]. In the Netherlands the incidence of MRSA infections is still quite low, probably as a result of the ‘search and destroy policy’, and the restricted use of antibiotics [6], [7]. Recently, the number of MRSA infections in the Netherlands has been gradually increasing which is a major cause of concern [8], [9].

In order to understand the population biology of *S. aureus* and to study the impact of measures to control MRSA infections unambiguous characterization of *S. aureus* isolates is required. Many typing techniques have been employed for the analysis of *S. aureus*. For many years phage typing was used [10], but with the advent of molecular typing, phage typing was replaced by newer techniques. A plethora of techniques has been used such as ribotyping [11], [12], random amplified polymorphic DNA analysis [12], [13], sequence analysis of 16S–23S rDNA *spa*cer regions [14], [15], amplified fragment length polymorphism [16], [17] and SSC*mec* typing [18], [19]. However, the most widely used molecular typing techniques for *S. aureus* are PFGE [20], [21], MLST [21], [22] and *spa*-sequence typing [23], [24]. In PFGE the fragments obtained after *Sma*I macrorestriction are separated on a special agarose gel. This has been extremely helpful in elucidating population structures and in the identification of *S. aureus* outbreaks. MLST, which is based on the DNA sequence analysis of 7 house keeping genes, yields unambiguous typing results that are suited for interlaboratory comparisons. This has resulted in an internet accessible database which is used world wide (http://saureus.mlst.net/). *Spa*-sequence typing has become a widely distributed typing technique for *S. aureus* in a very short time. In *spa*-sequence typing the variation in a tandem repeat region of the protein A encoding *spa* gene is utilized to perform genotyping of *S. aureus*. The repeats in the *spa* gene vary both in number and in sequence. By determining the sequence of the repeats, a profile is constructed which can be used for clustering. This has led to a fast growing internet accessible database which can be queried (http://www.spaserver.ridom.de/).

Like most other band-based genotyping methods, PFGE is slowly becoming an outdated technique. The method is not portable, sizing of bands is inaccurate leading to ambiguous results and the creation of profiles is labor intensive and difficult to perform. MLST is very expensive, labor intensive and therefore not available for most laboratories. *Spa*-sequence typing is a portable technique and is relatively easy to perform. However, it is based on a single locus in the genome only and clustering of *S. aureus* isolates based on *spa*-data is complex. For these reasons we developed and validated a typing technique based on the composition of genomic loci containing tandem repeats. This technique called multiple-locus variable-number tandem repeat analysis (MLVA) has been introduced as a typing method for a large number of bacterial pathogens [25]–[37]. In MLVA, the variability in the number of short tandem repeat sequences is utilized to create DNA profiles for epidemiological studies. Several MLVA schemes for *S. aureus* have been designed and used to type this pathogen. However, these MLVA schemes rely on analysis in agarose gels making them inaccurate and not portable. In this report we describe the development of a robust and portable MLVA and demonstrate the utility of this technique by identifying types and complexes in a large collection of methicillin-resistant and methicillin-susceptible *S. aureus* isolates.

## Materials and Methods

### Bacterial strains

In this study we included a collection of 2525 strains consisting of i) a test set of 86 *S. aureus*+10 *S. epidermidis* isolates, ii) a set of 1681 human and 100 pig-related *S. aureus* isolates used for extensive validation, iii) 658 *S. aureus* isolates used to assess the potential of MLVA to identify outbreaks. The test set included reference strains and was used for the initial set up of the MLVA. The isolates used for extensive validation were collected for the national *S. aureus* surveillance by the department of bacterial typing of the Laboratory of Infectious Diseases and Perinatal Screening of the National Institute for Public Health and the Environment. Of this strain set, 1781 were collected from 2005 to 2007 out of which 1681 strains were isolated from humans and 100 from pigs. For this study we used 393 isolates collected in 2005, 617 collected in 2006 and 671 isolates collected in 2007. The isolates were collected during the first 3 months in each of the 3 years and represented approximately 20% of the total number of isolates collected for the national surveillance during that time period. Of the 1681 strains from humans, 135 were isolated from blood, 831 originated from nasal, throat or perineum swabs and the remainder was predominantly isolated from wound infections. Approximately 87% of the strains isolated from humans and all strains isolated from pigs were methicillin-resistant *S. aureus* (MRSA). The collection of 1681 strains isolated from humans also included 163 strains that were part of the Dutch strains collected for the EARSS project (http://www.rivm.nl/earss/) 156 of which were methicillin sensitive *S. aureus* (MSSA). The 100 MRSA strains isolated from pigs were all collected in 2007. In addition, a set of 658 MRSA isolates collected for the national *S. aureus* surveillance in 2008 was used for a preliminary assessment of the epidemiological potential of the MLVA.

### Bacterial growth and preparation of lysates

Cultures stored at −80°C were streaked on Columbia agar plates with 5% sheep blood, cultured overnight at 37°C and visually inspected for purity. Two colonies were suspended in 50 µl lysis mix in TE (10 mM Tris. HCl, 1 mM EDTA, pH 8.0) supplemented with 100 µg/ml lysostaphine, incubated for 35 min at 37°C and heated for 10 min at 95°C. After the inactivation step 450 µl TE was added and the lysate was used either directly or stored at −20°C until use in PCR.

### Pulsed Field Gel Electrophoresis and *Spa*-sequence typing

Pulsed Field Gel Electrophoresis (PFGE) was performed as described before [38]. *Spa*-sequence typing was essentially performed according to the RIDOM protocol [24], (http://www.ridom.de) with slight modifications. The first modification was the use of a new forward primer (TAAAGACGATCCTTCAGTGAGC) to replace the *spa*-1113f primer used for *spa*-PCR and sequencing which has a sequence mismatch at the 16^th^ position with all known *spa* gene sequences. The reverse primer used was *spa*-1514r as described by the RIDOM protocol. Furthermore, we reduced the amount of primer used in the *spa*-PCR to minimize the amount of unused primer after PCR. As a result no purification of the PCR product was required for sequence analysis. Briefly, 1 µl of staphylococcal lysate was used in a 25 µl PCR using HotStar master mix (Qiagen, Hilden, Germany) and 2.5 pmol of primers. The *spa* gene was amplified using the following program: 15 min 95°C, 30 cycles of amplification that consisted of 45 sec at 95°C, 45 sec at 60°C, and 1.5 min at 72°C, and a final step of 7 min at 72°C. Two µl of the unpurified PCR product was used for sequencing both strands of the product.

### Identification and selection of VNTR loci in *S. aureus*

**Figure 1:**
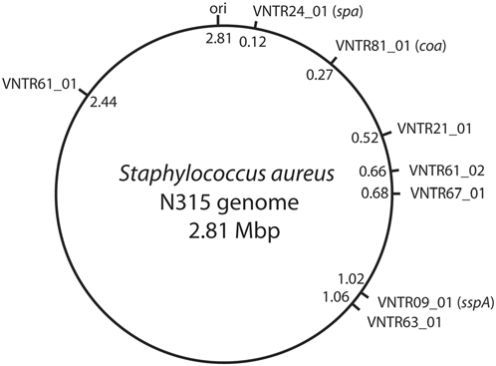
Schematic map of the genome of *S. aureus* strain N315, indicating the positions of the VNTR loci used in MLVA.

At the start of this study the genome sequences of 9 different *S. aureus* strains were available in the public domain. These genome sequences were screened for the presence of tandem repeat sequences using the Tandem Repeats Finder program, version 4.00 [39] and a home made script for the Kodon 3.5 software (Applied Maths, Sint-Martens-Latem, Belgium).The *in silico* analysis of the available *S. aureus* genomes revealed the presence of loci with a variable number of tandem repeated sequences in these sequenced genomes. Remarkably, the majority of the identified VNTR loci carried imperfect tandem repeats. Primers flanking the VNTR regions were designed and used on a randomly selected, genetically diverse test panel of strains that had previously been analyzed by PFGE in our laboratory. VNTR loci were considered as suitable if they yielded PCR products with all strains in the test panel and there were at least 3, but no more than 10 different alleles in the test set. Repeat loci that did not yield a single PCR product were considered unsuitable. Furthermore, VNTR loci that were located in non-coding regions in the genome were preferred, but this was not an exclusion criterion. Of the more than 20 VNTR loci that originally were identified by the *in silico* analysis 10 were selected based on their characteristics (repeat size, number of repeats, unique locus). Of these 10 we selected 8 VNTR loci that fulfilled the above mentioned criteria. Only 3 of the 8 loci were located in coding regions in the *S. aureus* genome (Table 1 and Fig. 1). The rationale for choosing 8 VNTR loci was pragmatic. Firstly, sufficient loci need to be incorporated to obtain enough discriminatory power. Secondly, the automated DNA sequencer used for the MLVA can distinguish 5 different fluorescent labels in one channel. This implies that a multiplex PCR with 4 different targets using a different fluorescent label for each PCR can be analyzed together with an internal marker with a fifth fluorescent label to obtain accurate sizing. Thus 2 multiplex PCRs enabled the simultaneous analysis of 8 different VNTR *S. aureus* loci.

**Table 1:** Properties of the VNTR loci used for MLVA of 1681 *S. aureus* isolates.

VNTR	Properties of VNTR locus	Average repeat size	No. of variable bases	DI^1^	Range no. repeats
VNTR09_01	*sspa*: cysteine protease	9	5	80.0	6–25
VNTR61_01	non-coding	60	2	79.5	0–6
VNTR61_02	non-coding	66	23	66.7	0–5
VNTR67_01	non-coding	67	0	78.9	0–10
VNTR21_01	non-coding	21	0	34.2	0–13
VNTR24_01	*spa*: protein A	24	7	83.0	1–18
VNTR63_01	non-coding	64	6	73.6	0–11
VNTR81_01	*coa*: staphylocoagulase	81	26	69.0	1–9

1 DI, Simpson's index of diversity.

### Multiple-locus Variable Number Tandem Repeat Analysis

VNTR PCRs were performed in 25-µl volumes in Applied Biosystems 9700 PCR machines (Applied Biosystems, Foster City, USA). Eight VNTR loci were amplified in 2 multiplex PCRs. For each multiplex PCR 2 µl of 1∶10 diluted *S. aureus* lysate was added to a mixture containing 10 pmol of each of 4 differently 5′ fluorescently labeled forward primers, 10 pmol of each of 4 unlabeled reverse primers and 12.5 µl of HotStarTaq multiplex-mastermix (Qiagen, Hilden, Germany). The exception was in the second multiplex PCR in which 5 pmol labeled VNTR61_02FV primer and also 5 pmol unlabeled primer VNTR61_02F were used to reduce the signal for this VNTR in the multiplex PCR. All primer sequences are shown in Table 2. VNTR loci were amplified using the following PCR program: 15 min at 95°C, followed by 20 cycles of amplification that consisted of 45 sec at 95°C, 45 sec at 54°C, and 90 sec at 72°C, and a final step of 30 min at 68°C to ensure complete terminal transferase activity of the Taq DNA polymerase. After PCR, samples were diluted 1∶100 in water and 1 µl of the diluted samples were mixed with 10 µl of 1∶100 in water diluted fluorescently labeled GeneScan 1200 LIZ-marker (Applied Biosystems). After heat denaturation for 5 min at 95°C, fragments were separated on an ABI 3730 DNA sequencer using the standard fragment analysis module. The resulting .fsa files were imported and analyzed in the GeneMarker software (Softgenetics, State College, USA) to calculate the number of repeats of each VNTR locus. VNTR loci that did not yield a PCR product after repeated analysis were assigned number 99. However, all 1681 isolates of the validation set yielded a PCR product for all VNTR loci. The assessed numbers of repeats of the 8 VNTR loci were combined into a string consisting of 8 integers e.g. 14-0-2-4-1-7-1-6. This string, referred to as the MLVA profile, was used for clustering.

**Table 2:** List of primers used for MLVA of *S. aureus*.

VNTR	Forward primer sequence^1^	Reverse primer sequence	Start^2^	End^2^
VNTR09_01	F-ATAAGCATTGAAACCATTATGATG	GCAACTTCTTAAAACAAAATATTG	1021501	1021864
VNTR61_01	N-AATGCACATGAAACACTAATT	GGTCAAGAATATTTAAAATCAATT	2440537	2440899
VNTR61_02	V-CTGTGAAGTTAGATAGATGAGTTT	GCAATTAACGATTTCTTCAC	659826	660093
VNTR61_02^3^	CTGTGAAGTTAGATAGATGAGTTT	GCAATTAACGATTTCTTCAC	659826	660093
VNTR67_01	P-CGTGAATCTCTTTTATAAGAGTGT	CCCTCCTATTAATATATATACCGT	680255	680600
VNTR21_01	V-GTCGATAAAGCATAAAGCTTT	AGCAATGAATCAATAATTTTCA	526755	526904
VNTR24_01	P-CAGCAGTAGTGCCGTT	GTAACGGCTTCATCCA	122873	123289
VNTR63_01	F-TGAAGATGTAGTAGGAATGTTAGT	AGAAAAAGCTAAAGAAGTTGAA	1056187	1056833
VNTR81_01	N-TTTGGATATGAAGCGAGA	CATATGTCGCAGTACCATC	266137	266632

1 Fluorescent dyes are indicated by a character. F, FAM; N, NED; V, VIC; P, PET.

2 Coordinates in NC_002745.

3 Unlabeled version of the primer VNTR61_02, used to reduce the fluorescence signal in the MLVA (see Materials and Methods).

The calculation of the number of repeats in each of the VNTR loci was straightforward. The size of the PCR product minus the size of the flanking regions yielded the repeat region. Dividing the size of this region by the size of the repeat unit yielded the number of repeats in each locus. This was true for all VNTR loci with the exception of VNTR61_01. The upstream flanking region of this locus turned out to be heterogeneous in composition, resulting is slightly larger or smaller fragments. Some of the bin sizes used in the GeneMarker software were shifted to fit the actual sizes of the various alleles which ensured accurate calculation of the number of repeats in the VNTR61_01 locus. Representatives of all allelic variants were sequenced to ensure the assignment of the number of repeats was correct.

### Data analysis

All typing data were imported into the Bionumerics software (Applied Maths), clustered using the appropriate settings and the relationships displayed using graphing method called minimum spanning tree as described before [34]. In the minimum spanning tree, the priority rule to first link types that have the highest number of single locus variants was chosen. In the tree, types are represented by circles. In our preferred settings, the size of a circle indicates the number of strains with this particular type. Heavy short lines connecting two types denote types differing in a single locus, thin longer lines connect double locus variants. MLVA complexes were created if the distance between neighboring types was not larger than 1 and at least 5 types fulfilled this criterion. For calculation of the genetic diversity and discriminatory ability of typing the Simpson's index of diversity was used, 
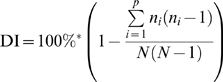
 where n_i_ is the number of strains belonging to i^th^ type and N is the total number of strains in the sample population [34], [40].

Statistical analyses were performed either in Bionumerics or in StatsDirect v2.6.6 (StatsDirect Ltd., Cheshire, UK). In Bionumerics the stability of the defined groups is calculated using a statistical method called group separation. The method uses the Jackknife method in which each entry is taken from the list and identified against each group. In this study this was done by using the average similarities of each group for identification.

### DNA sequencing

For DNA sequencing reactions, BigDye Terminator and BigDye XTerminator technologies were used (Applied Biosystems). Sequence reaction mixtures were analyzed on an ABI 3730 automated DNA sequencer.

## Results

### Design and development of MLVA for *S. aureus*

Based on the available *S. aureus* genome sequences VNTR loci were selected and evaluated for use in MLVA. Eight VNTR loci were selected and analyzed in 2 multiplex PCRs. Suitability was tested using a set of 96 strains which included 4 *S. aureus* isolates for which the genome has been sequenced (MW2, COL, N315, NCTC8325), 10 MRSA strains (5 pairs) considered as outbreak isolates based on their identical PFGE profiles, isolation date and the fact that they originated from the same hospital, 72 randomly selected MRSA isolates, 5 MSSA isolates and 5 methicillin resistant *Staphylococcus epidermidis* (MRSE) isolates and 5 methicillin sensitive *S. epidermidis* (MSSE) isolates. All *S. aureus* isolates yielded PCR products that varied in size. The strains from which the genome has been sequenced yielded the expected number of repeats in the various VNTR loci. Strains belonging to the same outbreak had identical MLVA profiles. None of *S. epidermidis* isolates yielded PCR products with any of the VNTR PCRs.

To determine the stability of the VNTR loci 5 *S. aureus* isolates were subcultured for 20 consecutive days by streaking a single colony from each strain on agar plates. Suspensions were made of each subculture and subjected to MLVA. None of the 8 VNTR loci were altered during the serial passage, showing that at least under laboratory conditions the VNTR loci were stable.

### MLVA of *S. aureus* isolates

**Figure 2:**
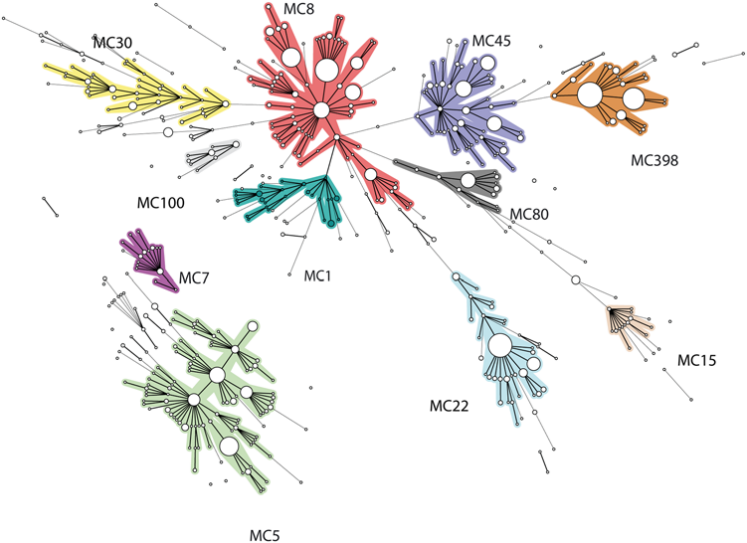
Minimum spanning tree of the 1681 *S. aureus* isolates typed by MLVA. Clustering of MLVA profiles was done using a categorical coefficient. In the minimum spanning tree the MLVA types are displayed as circles. The size of each circle indicates the number of isolates with this particular type. Thick solid lines connect types that differ in a single VNTR locus and a thin solid connects types that differ in 2 VNTR loci. The color of the halo surrounding the MLVA types denote types that belong to the same complex. MLVA complexes were assigned if 2 neighboring types did not differ in more than 1 VNTR locus and if at least 5 types fulfilled this criterion. MLVA complexes are also indicated in characters e.g. MC8 denotes MLVA complex 8.

MLVA was performed on 1681 *S. aureus* isolates originating from human patients. All isolates of this validation set yielded a PCR product for all VNTR loci. Analysis of the composition of the 8 VNTR loci of the *S. aureus* strain collection revealed that the diversity in the number of repeats varied among the various VNTR loci. Some loci carried large numbers of repeats e.g. up to 25 repeats in the VNTR09_01 locus, while other loci only carried a limited number of repeats e.g. VNTR61_02 with a maximum of 5 repeats (Table 1). The diversity indices (DI) of the 8 different VNTR loci also differed considerably. The lowest DI (34.2%) was found for VNTR21_01 and the highest was found for VNTR24_01 (83%), which is the *spa* gene (Table 1). In total 511 different MLVA types were found among the 1681 *S. aureus* isolates. The MLVA profiles were clustered using a categorical clustering coefficient and a minimum spanning tree was constructed to display the relationships between the various MLVA types (Fig. 2). This revealed the presence of 11 different MLVA complexes that were assigned complex names e.g. MC8 represents MLVA complex 8. Of the 1681 isolates used for the study 1473 (87.6%) were part of an MLVA complex. The remaining 208 isolates (12.4%) were not part of these complexes and were assigned a Nearest MLVA Complex (NMC) code e.g. NMC8 or 8, indicating the most closely related MLVA complex.

The majority of the isolates included in this study were MRSA (78.1%). The MSSA isolates were found in virtually all MLVA complexes. However, complexes MC100, MC15 and all 19 isolates that were closely related to MC15 (NMC15) were completely made up of methicillin sensitive *S. aureus*. In addition, only 7.7% of the isolates in MLVA complex MC7 were MRSA. In contrast, only 2 isolates (0.9%) in complex MC398 were MSSA (Table 3). For 1185 isolates the presence of the Panton-Valentine leukocidin (PVL) genes was also determined [41] and this revealed that 94.2% of the isolates in MC80 were PVL-positive (Table 3). The other 2 MLVA complexes that contained a considerable number of PVL-positive isolates were MC30 (34.4%) and MC8 (17.8%).

**Table 3:** Characteristics of MLVA complexes found among 1681 *S. aureus* isolates.

MLVA complex	No. of isolates	% of isolates	No. of types	DI^1^	% mecA+	% PVL+
MC8	371	22.1	67	90.9	86.0	17.8
MC5	278	16.5	85	93.4	90.3	2.7
MC398	216	12.8	22	72.1	98.6	1.4
MC45	203	12.1	47	91.3	76.9	0.0
MC22	176	10.5	30	76.8	92.1	2.4
MC30	68	4.0	35	95.5	60.3	34.4
MC80	55	3.3	12	67.1	90.9	94.2
MC1	42	2.5	26	95.8	59.5	0.0
MC7	26	1.5	15	92.6	7.7	0.0
MC100	20	1.2	8	83.2	0.0	0.0
MC15	18	1.1	13	96.1	0.0	0.0
No complex	208	12.4	151	99.2	45.2	14.9
total	1681	100	511	98.5	78.2	11.3

1 DI, diversity index.

### Comparison of MLVA with *spa*-sequence typing and PFGE

**Figure 3:**
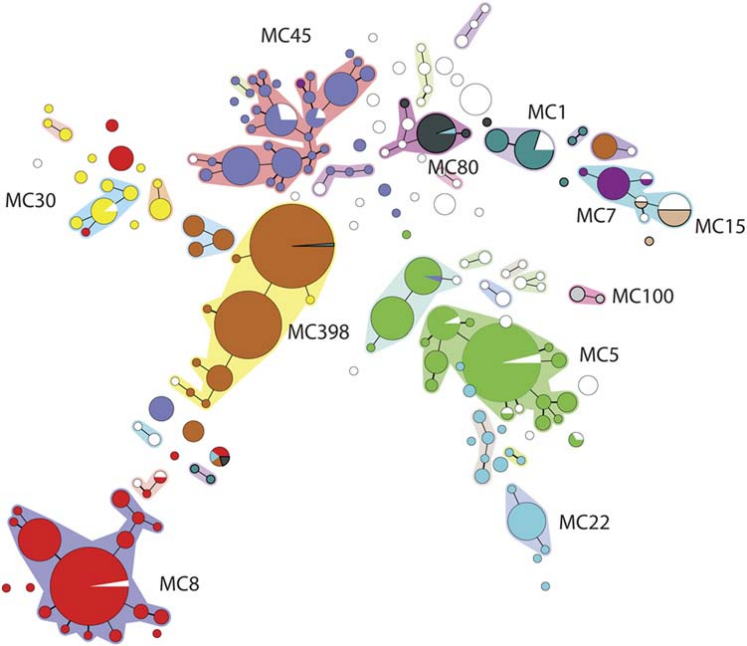
Minimum spanning tree of 882 *S. aureus* isolates typed by *spa*-sequence typing. The SpaTypes are displayed as circles. For description of the symbols, lines etc. see legend of figure 2. Clustering was performed using the Bionumerics spa-plugin with a conservative alignment setting of 100% and 0% maximum duplication length. Halos surrounding SpaTypes indicate related SpaTypes.

**Figure 4:**
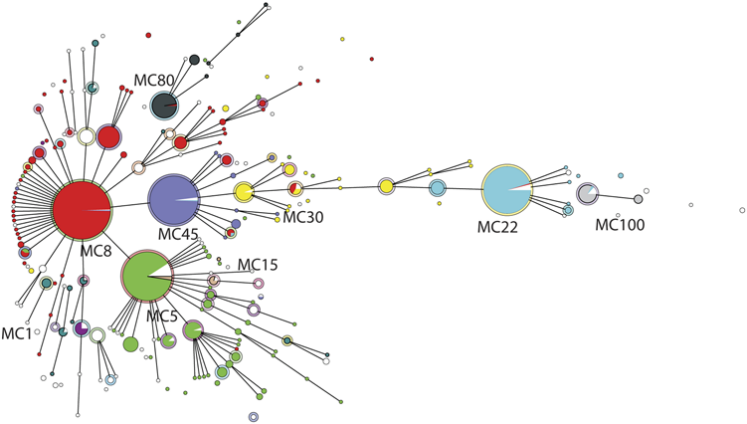
Minimum spanning tree of 1304 *S. aureus* isolates typed by PFGE. The PFGE types are displayed as circles. Clustering was performed using the Pearson correlation coefficient and a 2% optimization setting. The resulting similarity matrix was used to construct a minimum spanning tree using a 10% similarity bin size. Related PFGE grouped and this is denoted by the double circle (compacted complexes). The MLVA complex is denoted as the color of the circles (see figure 2) and is also indicated in characters.

A large number of isolates previously characterized using *spa*-sequence typing and/or PFGE was used to assess the value of MLVA as a typing method for *S. aureus*. *Spa*-sequence typing had been performed for 882 of the 1681 isolates. SpaTypes were clustered using the *spa*-plugin in the Bionumerics software and the results were displayed in a minimum spanning tree. The minimum spanning tree (Fig. 3) showed that the grouping by *spa*-sequence typing was similar to the grouping found by MLVA. One of most notable findings was that isolates belonging to MC7 and MC15 resided in the same *spa* cluster. PFGE analysis had been performed on 1304 of the 1681 isolates. Again clustering based on PFGE yielded grouping that was similar to that of the MLVA (Fig. 4). However, due to small inaccuracies inherent to the experimental variations of PFGE, grouping was not as clear cut as with MLVA or *spa*-sequence typing.

To quantify the degree of similarity in grouping obtained by the 3 methods, both congruence between the typing methods and group separation were determined using Bionumerics software. Congruence of typing methods is derived from the similarity matrices that are made in the analyses of the methods. Analysis using a Pearson correlation coefficient revealed that the congruence between *spa*-sequence typing and MLVA amounted 62.7% for the set of 882 isolates that were typed by both methods. The congruence between PFGE and MLVA performed on 1304 isolates was significantly lower and amounted 48.0%. Group separation was calculated by the Jackknife algorithm. In this case groups represented the 11 MLVA complexes. The analysis showed that 95.1% of the isolates belonged to these 11 groups if *spa*-sequence typing was used for identification. Group separation was 89.2% if PFGE was used to characterize the isolates. This indicates that there is considerable degree of similarity in grouping using the 3 typing methods.

### Diversity indices of MLVA, PFGE and *spa*-sequence typing

**Figure 5:**
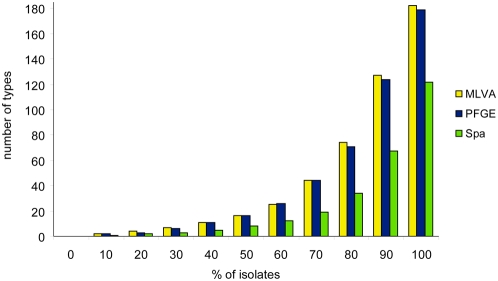
Discriminatory power of MLVA, *spa*-sequence typing and PFGE for *S. aureus.* Diversity is plotted as the number of types versus the percentage of isolates. To obtain the plot the list of typing results was first sorted by frequency and the percentage of isolates versus the number of types was determined. The plot only shows the results for the 529 *S. aureus* isolates that were analyzed by all 3 methods.

Diversity indices can be used to assess the diversity of a bacterial population. However, the diversity index is also often used to indicate the discriminatory power of typing methods. Simpson's diversity index was determined for all isolates typed with any of the 3 methods and for the 529 isolates for which all 3 typing methods had been performed (Table 4). This revealed that MLVA and PFGE yielded similar DIs of 98.5% and 97.7%, respectively. The DI of the *spa*-sequence typing was 96.2% and thus somewhat lower than those of MLVA and PFGE. This would suggest a relatively small difference between the three typing methods. However, if the number of types is plotted against the cumulative proportion of isolates in the sampled population a more pronounced difference between MLVA, PFGE and *spa*-sequence typing emerges (Fig. 5). The plot revealed a considerable difference in discriminatory power of MLVA and PFGE as opposed to *spa*-sequence typing. In the collection that was sorted by type frequency, 70% of all isolates represent 44 MLVA types and 44 PFGE types. In contrast, 70% of all isolates represent only 19 SpaTypes, less than half the number of types obtained by MLVA and PFGE. The higher discriminatory power of MLVA is also apparent from the fact that there are significantly more isolates per type for *spa*-sequence typing than for MLVA (table 4).

**Table 4:** Diversity indices of the genotyping methods for all isolates typed with any of the 3 methods and for 529 isolates typed with all 3 methods.

	No. of isolates	No. of types	No. of isolates per type	DI (95% CI)^1^
All typed isolates				
MLVA	1681	511	3.3	98.5% (98.3%–98.7%)
*Spa*	882	192	4.6	96.2% (95.7%–96.7%)
PFGE	1304	321	4.1	97.7% (97.4%–98.0%)
Typed with all 3 methods				
MLVA	529	182	2.9	98.2% (97.9%–98.5%)
*Spa*	529	122	4.3	94.9% (94.0%–95.9%)
PFGE	529	179	3.0	97.9% (97.5%–98.3%)

1 DI, diversity index; 95% CI, 95% confidence interval.

### Relationship between MLVA and MLST

Four lines of observations support the notion of a strong agreement between the groupings generated by MLST and MLVA. (1) Direct comparison between MLST and MLVA based on 40 isolates, (2) indirect comparison of MLVA with MLST based on the data derived from the Ridom database, (3), circumstantial evidence that *pvl* genes were only present in MC8, MC30 and MC80, (4) *in silico* analysis of congruence using 14 fully sequenced genomes.

**Figure 6:**
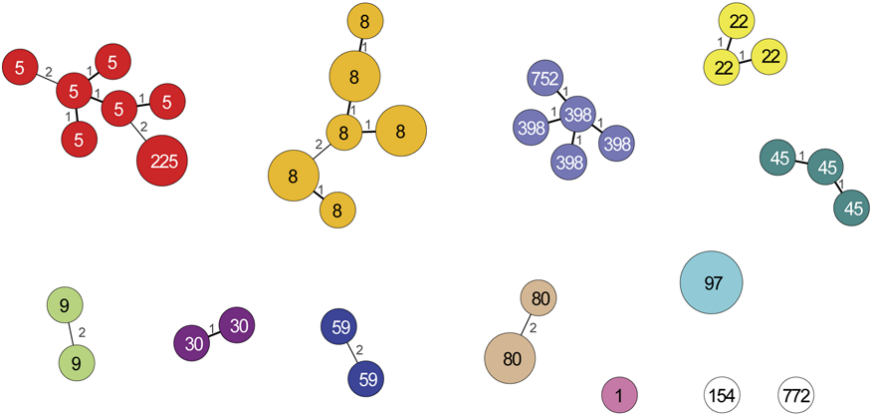
Concordance of the grouping obtained by MLVA and by MLST. A minimum spanning tree was constructed based on MLVA data of 40 isolates for which MLST had already been performed. The circles represent the MLVA types and the numbers in the circles represent the STs. The numbers displayed between types denote the number of VNTR loci that differ between these types. The colors represent the MLST clonal complexes. The white circles are STs that have not been assigned to a particular clonal complex. Only single locus or double locus MLVA variants are connected by lines. The size of each circle indicates the number of isolates with this particular MLVA type.

The agreement between MLVA and MLST was apparent from the comparison of the MLVA with MLST results obtained for a small set of 40 isolates for which we also had MLST data available that had previously been generated in our department. This revealed that MLVA was more discriminating than MLST, but more importantly that only STs belonging to the same MLST clonal complex were grouped by MLVA (Fig. 6)

More evidence for the agreement between MLVA and MLST was obtained in an indirect manner. An inventory was made on the *spa*-types that were present in the various MLVA complexes. After that the Ridom *Spa*-Server (http://spaserver2.ridom.de/mlst.shtml) was interrogated to reveal the relationship between *spa*-type and sequence type (ST). This revealed a remarkable agreement between the MLVA complexes and the MLST clonal complexes. With exception of one, each MLVA complex corresponded to a particular MLST clonal complex. For this reason the MLVA complexes were named in analogy to the MLST clonal complexes e.g. MLVA complex MC8 is made up of isolates that have *spa*-types belonging to MLST clonal complex CC8. There was a large MLVA complex, MC398, which could not be identified as corresponding to a known MLST clonal complex. This MLVA complex contained all pig MRSA isolates and was therefore named after the dominant pig MRSA sequence type ST398.

The strong agreement between MLVA and MLST was further supported by the observation that virtually all PVL-positive isolates were found in MC80, MC30 and MC8. This is consistent with the finding that most PVL-positive *S. aureus* isolates belong the MLST clonal complexes CC80, CC30 and CC8 [42].

**Figure 7:**
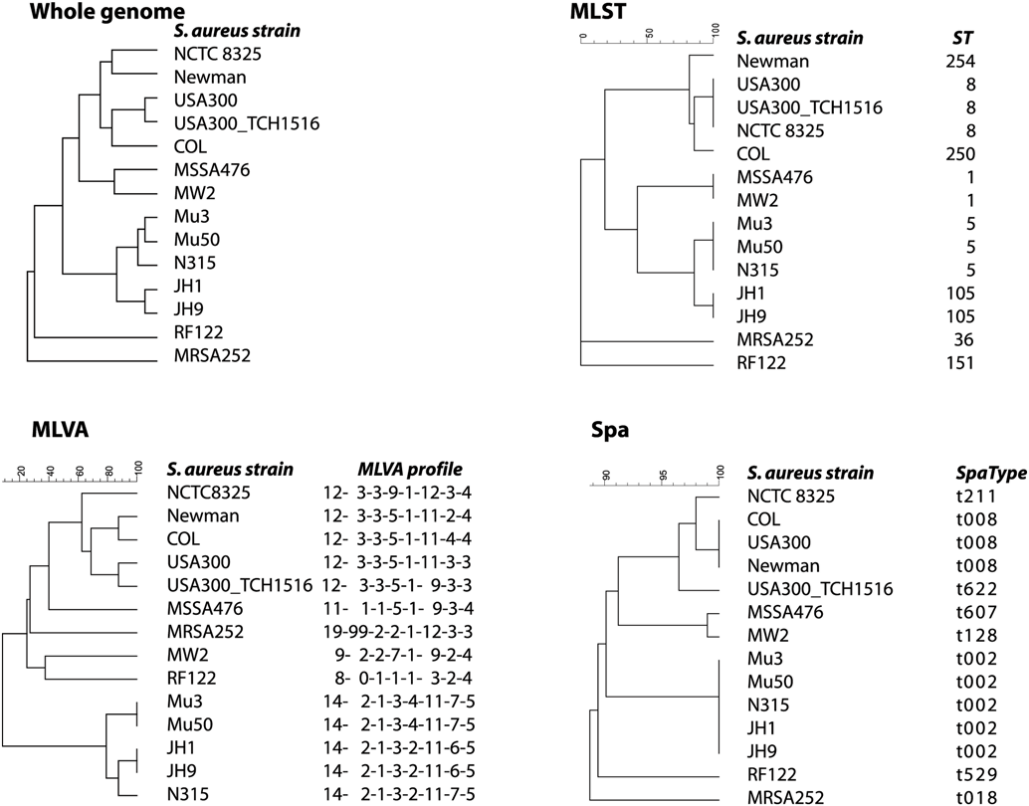
*In silico* analysis of 14 *S. aureus* strains for which the genome sequence has been determined. Strains were clustered on basis of their whole genome sequence, MLST profile, MLVA profile and *spa*-sequence type, respectively.

In an additional effort to corroborate the possible strong relationship between MLVA and MLST an *in silico* analysis of 14 sequenced *S. aureus* genomes was performed. The sequence types, MLVA profiles and *spa*-types were derived from the genomes sequences, clustered and subsequently congruence was calculated (Fig. 7). This showed that there was a 86.5% congruence between MLST and MLVA. The 66.7% congruence between MLST and *spa*-sequence typing was considerably lower than that between MLST and MLVA. For this data set congruence between MLVA and *spa*-sequence typing was 71.9%. The data set obtained from these 14 isolates is too small to draw firm conclusions. However, the high congruence between MLST and MLVA of this *in silico* data set suggests a high degree of similarity between MLST and MLVA based clustering.

### MLVA of pig MRSA isolates

**Figure 8:**
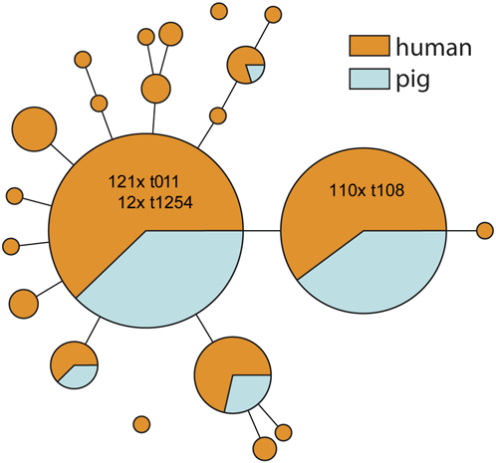
Minimum spanning tree of 295 pig MRSA isolates typed by MLVA. Each circle represents an MLVA type. The orange circles or sectors of circles denote types obtained from 195 pig MRSA strains isolated from humans. The MLVA profiles of 100 pig MRSA strains isolated from pigs are indicated in light blue. The numbers in the circles denote the SpaTypes and their frequency within the 2 dominant MLVA types.

Approximately 12% of the strains isolated from humans that were used for this study were screening cultures from community origin i.e. no hospital exposure either abroad or in the Netherlands which were taken because of exposure to pigs. These so called pig MRSA belonged to a single MLVA complex, MC398. Variation within this complex is very low with a diversity index of only 72.1%. Using MLVA this set of 195 isolates was compared to a set of 100 pig MRSA isolated from pigs (Fig. 8). As anticipated, MLVA could not discriminate strains isolated from humans from those isolated from pigs. There were 2 dominant MLVA types that represented the 2 dominant *spa*-types t011 and t108. Isolates with SpaType t1254 were of the same MLVA type as those with SpaType t011. Considering there is only a single base pair difference between t011 and t1254 such similarity is not surprising. The difference between the dominant MLVA types representing isolates with SpaTypes t011 and t108 is caused solely by the presence of an extra *spa* repeat (r34) in t011. This extra repeat results in a single locus difference between the 2 major MLVA types.

### Potential of MLVA to identify outbreaks

After validating the technical performance of the MLVA in our lab, all isolates submitted as part of the national MRSA surveillance in 2008 were characterized by *spa*-sequence typing and MLVA. This allowed us to make a preliminary assessment of the epidemiological concordance of the MLVA among isolates from patients with proximal temporal and spatial relationship. A selection of 658 isolates from hospital laboratories that submitted 30 or more isolates between January and October was included. Space-time clusters were defined as the isolation of 3 or more primary isolates from individual patients treated in the same hospital within a 2 month window displaying the same SpaType . This revealed the occurrence of 38 space-time SpaType clusters in the 15 hospital laboratories (Table 5). In 29 of the 38 clusters (76%) only a single MLVA type was found within the cluster. However, in 9 of the 38 clusters (24%) each cluster contained 2 or 3 MLVA types. In some clusters the deviating MLVA type represented a single locus MLVA variant (e.g. 1^st^ cluster in May-Jun, hospital B). This may represent a change of the MLVA profile within the same strain. However, several other Spa-clusters yielded isolates with MLVA profiles that differed in 3 loci (e.g. 2^st^ cluster in May-Jun, hospital B). It seems rather unlikely that the isolates that disagree in three unlinked genetic loci can be regarded as epidemiologically related. It is far more likely that *spa*-sequence typing failed to differentiate these isolates and thus incorrectly suggest epidemiological relatedness. This preliminary study shows that MLVA may be more reliable in identifying clusters of epidemiologically related isolates which may represent an outbreak of MRSA. However, a carefully planned study with detailed data on patient movements is required to assess the final validity of MLVA as a molecular epidemiological tool.

**Table 5:** Space-time SpaType clusters found in isolates collected from 15 hospitals during the time period January–October 2008.

Hospital	Isolates	Episode	Cluster size	SpaType	MT^1^	No. with MT	Spa repeats	MLVA profile
A	38	May–Jun	8	t038	527	8	08-39-34-34-13-17-34-16-34	11-02-03-04-01-10-01-05
B	57	Jan–Feb	5	t447	67	5	26-23-34-17-20-17-12-17-16	14-02-01-03-02-10-06-05
			3	t008	314	3	11-19-12-21-17-34-24-34-22-25	12-03-03-05-01-11-02-03
		Mar–Apr	9	t179	5	9	26-23-17-34-17-20-17-12-12-16	14-02-01-03-02-11-06-05
		May–Jun	10	t002	5	9	26-23-17-34-17-20-17-12-17-16	14-02-01-03-02-11-06-05
				t002	86	1	26-23-17-34-17-20-17-12-17-16	14-02-01-03-02-11-05-05
			3	t179	5	2	26-23-17-34-17-20-17-12-12-16	14-02-01-03-02-11-06-05
				t179	776	1	26-23-17-34-17-20-17-12-12-16	14-02-01-03-01-11-01-04
		Jul–Aug	4	t179	5	4	26-23-17-34-17-20-17-12-12-16	14-02-01-03-02-11-06-05
C	31	Mar–Apr	3	t002	5	3	26-23-17-34-17-20-17-12-17-16	14-02-01-03-02-11-06-05
D	35	Jan–Feb	3	t064	195	3	11-19-12-05-17-34-24-34-22-25	12-02-02-05-01-11-02-04
E	56	Mar–Apr	3	t008	274	3	11-19-12-21-17-34-24-34-22-25	09-03-03-05-01-11-01-04
		May–Jun	17	t008	274	14	11-19-12-21-17-34-24-34-22-25	09-03-03-05-01-11-01-04
				t008	314	2	11-19-12-21-17-34-24-34-22-25	12-03-03-05-01-11-02-03
				t008	8	1	11-19-12-21-17-34-24-34-22-25	12-03-03-05-01-11-02-04
F	71	Jan–Feb	3	t026	545	3	08-16-34	11-03-02-06-01-04-01-05
		May–Jun	4	t026	545	3	08-16-34	11-03-02-06-01-04-01-05
				t026	511	1	08-16-34	11-03-02-04-01-04-01-05
		Jul–Aug	4	t003	130	4	26-17-20-17-12-17-17-16	12-02-01-03-02-09-06-05
			6	t065	980	3	09-02-16-34-13-17-34-16-34	11-04-03-02-01-10-01-05
				t065	988	2	09-02-16-34-13-17-34-16-34	11-04-03-04-01-10-01-05
				t065	1407	1	09-02-16-34-13-17-34-16-34	11-02-03-02-01-10-01-05
G	38	May–Jun	5	t1234	427	5	07-23-12-12-34-34-34-33-34	12-00-01-01-01-10-04-09
		Jul–Aug	4	t040	536	4	09-02-16-13-17-34-16-34	11-03-03-04-01-09-01-05
			3	t1416	540	3	09-02-16-16-34	11-03-03-04-01-06-01-05
H	33	Jan–Feb	3	t008	8	3	11-19-12-21-17-34-24-34-22-25	12-03-03-05-01-11-02-04
		May–Jun	4	t008	8	4	11-19-12-21-17-34-24-34-22-25	12-03-03-05-01-11-02-04
I	40	Jul–Aug	5	t223	491	5	26-23-13-23-05-17-25-17-25-16-28	18-05-03-01-01-12-01-05
J	31	Sep–Oct	4	t008	314	4	11-19-12-21-17-34-24-34-22-25	12-03-03-05-01-11-02-03
			3	t334	303	3	11-12-21-17-34-22-25	12-03-03-01-01-08-02-04
K	60	May–Jun	3	t008	269	3	11-19-12-21-17-34-24-34-22-25	09-03-03-05-01-99-02-04
			3	t045	116	3	26-17-20-17-12-17-16	14-01-01-03-02-08-06-05
L	35	Mar–Apr	7	t045	116	7	26-17-20-17-12-17-16	14-01-01-03-02-08-06-05
		May–Jun	9	t045	116	9	26-17-20-17-12-17-16	14-01-01-03-02-08-06-05
			3	t267	632	3	07-23-12-21-17-34-34-34-33-34	12-00-01-01-01-11-04-07
M	30	Mar–Apr	9	t038	527	9	08-39-34-34-13-17-34-16-34	11-02-02-04-01-10-01-05
N	45	Jan–Feb	9	t740	528	9	08-39-34-13-17-34-16-34	11-02-03-04-01-09-01-05
		Mar–Apr	8	t740	528	8	08-39-34-13-17-34-16-34	11-02-03-04-01-09-01-05
		May–Jun	5	t740	528	5	08-39-34-13-17-34-16-34	11-02-03-04-01-09-01-05
		Jul–Aug	3	t008	240	3	11-19-12-21-17-34-24-34-22-25	12-03-03-05-01-11-01-04
			5	t740	528	5	08-39-34-13-17-34-16-34	11-02-03-04-01-09-01-05
		Sep–Oct	3	t008	240	3	11-19-12-21-17-34-24-34-22-25	12-03-03-05-01-11-01-04
O	58	Jan–Feb	4	t008	8	4	11-19-12-21-17-34-24-34-22-25	12-03-03-05-01-11-02-04
		May–Jun	21	t064	836	12	11-19-12-05-17-34-24-34-22-25	12-02-02-05-02-11-06-05
				t064	195	6	11-19-12-05-17-34-24-34-22-25	12-02-02-05-01-11-02-04
				t064	189	3	11-19-12-05-17-34-24-34-22-25	12-02-02-05-01-11-01-04
			6	t3365	194	6	11-19-12-36-34-24-34-22-25	12-02-02-05-01-10-02-04
		Sep–Oct	12	t064	195	12	11-19-12-05-17-34-24-34-22-25	12-02-02-05-01-11-02-04

1 MT, MLVA type.

## Discussion

In this study we validated a newly designed MLVA using a collection of 1681 *S. aureus* strains isolated from Dutch patients. The MLVA was compared with previously obtained PFGE and *spa*-sequence typing data and this revealed that MLVA was at least as discriminatory as PFGE, yielding 511 different MLVA types, twice as many types as *spa*-sequence typing did. MLVA yielded unambiguous numerical profiles representing the number of repeats present in 8 different VNTR loci in the *S. aureus* genome. The MLVA profiles, strings of 8 integers, were used for categorical clustering of isolates analyzed in this study. The clustering yielded 11 distinct groups, which we designated as MLVA complexes. Some of these complexes were predominantly made up of MSSA only, while other groups almost exclusively consisted of MRSA. At the typing level there was moderate typing congruence between MLVA, *spa*-sequence typing and PFGE. The congruence between these methods was much higher at the group level with group separation values of 95% for the relationship between MLVA and *spa*-sequence type grouping and 89% for that of MLVA and PFGE. Analysis of a set of 40 isolates that had been previously typed by MLST supports a high degree of concordance between MLVA and MLST. In addition, using available *spa*-sequence typing data, we were able to show that there was a high agreement of MLVA complexes with MLST clonal complexes. However, systematic comparison between MLVA and MLST will have to be performed to corroborate the deduced high agreement. Preparations for such comparison are currently in progress.

In recent years a possible new reservoir for MRSA has emerged in the Netherlands. There have been reports that the nasal carriage with MRSA has increased in pig farmers, and that these lineages of *S. aureus* are shared by farmers and their animals [43]–[45]. This pig-related MRSA appears to be clonal and identified by MLST as sequence type 398 [8], [46]. MLVA of these ST-398 strains isolated from humans and from pigs was utilized to assess possible differences between the human and animal isolates. However, such differences were not detected and MLVA revealed the same degree of differentiation as *spa*-sequence typing did. There were 2 major MLVA types that represented the 2 dominant *spa*-types t011 and t108 which differ in a single repeat.

To assess the potential of MLVA as a molecular epidemiological tool, MRSA submitted as part of the national surveillance strategy in 2008 were scanned for time–space clusters with indistinguishable SpaTypes. Thirty-eight were identified in this set of 658 isolates of which 76% were confirmed by identical clusters MLVA.profiles. Moreover, in 24% of the clusters, MLVA improved the resolution and discriminated further between the SpaTypes. This may be due to micro-evolution of the VNTR loci, but in instances where 2 or 3 VNTR loci differed it seems more likely that *spa*-sequence typing incorrectly grouped strains that were diverse at different chromosomal locations. These findings are an indication that MLVA can the enhance micro-epidemiological accuracy crucial for the understanding of the dissemination of MRSA in defined hospital settings. However, additional investigations supported by detailed patient contact studies are needed to appraise the full potential of this method as a microepidemiological typing tool.

Many different genotyping methods have been used to study the epidemiology of *S. aureus*, and for many years PFGE has been the method of choice because of its discriminatory power and relative simple equipment required to create PFGE profiles [20], [21]. However, the major disadvantage of PFGE is its low portability making it unsuitable for interlaboratory comparisons. The development of an MLST scheme for *S. aureus* has proven to be a major improvement [22]. Its portability and the unambiguous nature of the results probably make MLST the best choice for epidemiological studies population analyses. However, the high costs associated with sequencing 7 housekeeping genes for MLST have limited its use. The introduction of the *spa*-sequence typing has been quite successful [24]. In *spa*-sequence typing, sequencing part of only a single gene is required. This makes it a portable method with unambiguous results that is much more cost effective method than MLST. The major drawback of *spa*-sequence typing is that only a single locus in the genome is studied and a small change, even a single mutation, already yields a different type. It is difficult to interpret the meaning of such a change in its evolutionary context and in absence of more global view of the rest of the genome. *S. aureus* isolates that differ in one or more bases in the *spa* locus or even in the number of *spa* repeat units may represent closely related strains or very distant ones. The use of multiple genomic loci such as in MLST provides a more robust approach. The relationship between two isolates that differ in only one out of seven loci is much more obvious. At the same time increasing the number of loci also improves the discriminatory ability of any typing system. Like MLST, MLVA utilizes several genomic loci for genotyping.

The MLVA scheme presented here is not the first MLVA for *S. aureus* reported. Sabat et al. were the first to describe an MLVA based on 5 different VNTR loci [47]. They analyzed 34 isolates and detected 26 different types and they concluded that their MLVA was as discriminating and as reproducible as PFGE. However, their MLVA contained VNTRs with a repeat size of only 9-bp..which makes an accurate sizing of the bands on agarose gels impossible. Furthermore, PCR on some of the loci included in their MLVA e.g. the *sdr* locus, yielded multiple anonymous PCR products which compounded that PCR products could not be assigned to individual VNTR loci. Thus this MLVA scheme was unable to address genetic relationships. Typing results based on anonymous banding pattern resolved in agrose gels are – like PFGE - not portable and do not provide unambiguous results. Other groups have used the typing scheme of Sabat et al. or a slightly altered scheme [48]–[52]. Hardy et al. [53], [54] described the use of VNTRs which they, for reasons not entirely clear, designated staphylococcal interspersed repeat units (SIRUs). Again PCR products were sized on an agarose gels to calculate the number of repeats per locus and in the first study analysis of 16 *S. aureus* isolates yielded 11 different types. In the second study 116 isolates yielded 18 different SIRU profiles. Recently, Ikawaty et al. [55] reported the application of a slightly adapted version of Hardy's MLVA. The studied the polymorphism in 5 VNTR loci using a collection of 150 isolates and found 76 different MTs. Part of their study was performed using isolates from 6 outbreaks. However, these outbreaks were merely identified on the basis of identical phage typing and PFGE profiles but lacked any epidemiological corroboration. Remarkably, the MLVA profiles showed heterogeneity in 4 of the 6 reported outbreaks.

Using 2 multiplex PCRs, MLVA of 96 isolates can easily be performed within a single day provided a sequencing facility is readily available. In our laboratory setting, the cost for consumables for MLST analysis of a single strain amounts to approximately € 70. The costs for *spa*-sequence typing are much lower at € 9 per isolate and those for MLVA are even lower and amount to € 8. This makes MLVA nearly a factor 9 cheaper than MLST. A major advantage of MLVA over *spa*-sequence typing is the straightforward clustering of MLVA profiles compared to that of SpaTypes. In the MLVA clustering we used in this study, the distance between types is expressed as the number of loci that is different and the difference in the number of repeats is not taken into account. The complex algorithm used for clustering of SpaTypes is called ‘based upon repeat pattern’ (BURP) [56], [57]. It uses many different parameters such as repeat-duplication, repeat-excision and repeat-substitution, but also base-insertion and base-deletion events were used to calculate the relatedness of different SpaTypes. Furthermore, it excludes SpaTypes that are shorter than 5 repeats. The validity of using these parameters is uncertain because the mechanism that drive variation of that locus and the evolutionary clock are unknown. MLVA clustering is much simpler and is similar to the method used for clustering MLST profiles. In MLVA, loci are either identical or different, and it does not take into account the number of repeats that is lost or gained.

The study present here has been performed to validate MLVA as a new typing method for *S. aureus* and MRSA in particular. Van Belkum et al. [58] recently published an overview in which they provided criteria for the validation of a typing method. In our study we have demonstrated that MLVA fulfills all performance criteria described in Van Belkum's publication. The stability of the marker under laboratory conditions; there were no non-typeable isolates among the 2515 *S. aureus* isolates included in the study. The test population chosen for this study is well defined and probably comprises the largest collection ever used for such an analysis. Furthermore MLVA has been shown to be highly discriminatory and the typing has been shown to be reproducible and accurate which was corroborated by the sequence analysis of all allelic variants. The epidemiological validity has been addressed in a preliminary assessment by analyzing isolates with clear time-space relationship. MLVA was highly concordant and may even outperform *spa*-sequence typing, although a more detailed study will be required to corroborate this conclusion. Apart from the performance criteria, the MLVA described in this study also meets virtually all convenience criteria. Although the primers for this MLVA are designed for use with *S. aureus* only and cannot be used for other species the method itself is flexible and can be used for any bacterial species that carries VNTR loci. MLVA is fast, easy to use and highly suitable for high throughput typing against relatively low costs. It does not need exotic reagents or equipment and therefore is accessible for any laboratory that is equipped with PCR machines and an automatic DNA sequencer. Because of its straightforward computerized analysis, which leads to unambiguous numerical profiles, the data is highly suitable for the use in electronic databases.

In conclusion, the MLVA described in this study is a high throughput, relatively low cost genotyping method that yields unambiguous data that can be used for interlaboratory comparisons and for internet accessible databases. Currently we are developing a web platform in which it will be possible to query a database with all known MLVA profiles ensuring a uniform nomenclature for users of the method. This website will have a functionality that is similar to that found at the MLST website (www.mlst.net). The high throughput and the observed similarity with MLST grouping make MLVA a valuable typing technique that may enable the study the epidemiology of *S. aureus* at various geographic levels and we are confident that its true value will become obvious in detailed epidemiological studies.
